# Cost-Effectiveness and Effects of a Home-Based Exercise Intervention for Female Caregivers of Relatives with Dementia: Study Protocol for a Randomized Controlled Trial

**DOI:** 10.3390/healthcare8010054

**Published:** 2020-03-06

**Authors:** Miguel Madruga, Josué Prieto, Paloma Rohlfs, Narcís Gusi

**Affiliations:** 1 Physical Activity and Quality of Life Research Group, Faculty of Sport Sciences, University of Extremadura, 10071 Cáceres, Spain; josueprieto@usal.es (J.P.); palomaroh@unex.es (P.R.); ngusi@unex.es (N.G.); 2 School of Tourism and Education, University of Salamanca, 05003 Ávila, Spain; 3 Faculty of Nursing and Occupational Therapy and Faculty Training College, Department of Psychology and Anthropology, University of Extremadura, 10071 Caceres, Spain; 4 CIBER of Frailty and Healthy Aging (CIBERFES), 28089 Madrid, Spain; 5 International Institute for Innovation in Aging, 10071 Caceres, Spain

**Keywords:** home-based exercise, informal caregiver, cost-effectiveness, quality of life, fitness, psychological symptoms, burden

## Abstract

Previous research has proposed a range of support interventions to mitigate the adverse impact of caregiving on the daily life of caregivers of relatives with dementia. However, the effectiveness of these interventions shows a high variability. Informal caregivers usually lack the time and/or live too far from conventional facilities to do physical exercise, especially those who live in rural areas. Thus, home-based interventions may be more efficacious due to their greater convenience for this population. The present work proposes and describes a study protocol for a randomized control trial that will analyze the efficacy and cost-effectiveness of a home-based, structured individual physical exercise intervention to improve the health-related quality of life and the mental health of female informal caregivers of relatives with dementia. The nine-month-long intervention will comprise participation in two supervised physical exercise sessions per week at the caregiver’s home. The proposed study outcomes will be: (1) feasibility of and adherence to the home-based provision of the intervention; (2) improvement in physical fitness and quality of life; and (3) reduction in subjective burden, psychological symptomatology and depression. Analyses will also be performed to determine the cost-effectiveness after the intervention. In conclusion, this intervention might thus represent a tailored and feasible strategy to help caregivers cope with the physical and psychological stress resulting from caregiving-related responsibilities, and it could represent a novel cost-effective support home-based intervention for caregivers.

## 1. Introduction

Previous research has shown that about 46.8 million individuals had dementia worldwide in 2015, and that this amount will peak at 74.7 million and 131.5 million in 2030 and 2050, respectively [1]. Therefore, and in accordance with this trend, it is expecting that the number of required caregivers to assist patients with dementia in the world will also significantly increase [2].

Spain has one of the highest rates of dementia worldwide. Moreover, the prevalence of the disease in this country significantly varies with the age of the population: between 5.4% and 14.9% in subjects who are aged 65 years or older and between 7% and 19.2% in 70-year-old subjects or older [3]. 

Most individuals with dementia are cared for at home by an unpaid informal caregiver [4,5]. In the majority of cases, the informal caregiver is a female family member, typically the spouse or daughter, who is aged between 45 and 65 years, who resides at the home of the care-recipient [2,6,7,8].

Although the role of caregiver may be perceived as satisfying and rewarding [9], the progressive and protracted deterioration of the state of health of individuals with dementia [8,10] requires caregivers to provide them with continuous support in all areas of life for long periods of time [11]. Moreover, the exact length of these long periods, as well as all the things that may happen to the subject with dementia, is usually unknown by informal caregivers. As a consequence, the exercise of the role of the caregiver leads to a wide variety of physical, psychological, social and financial difficulties [7,12,13,14,15]. In this regard, the adverse effects on physical health and quality of life are diverse [16,17,18,19,20]. Informal caregivers show higher rates of hypertension and other cardiovascular-related disorders [21,22], as well as lower aerobic endurance [23] than non-caregivers. Informal caregivers have reported having severe back pain due to alterations in the trunk muscle extensor strength [8,20], and other osteomuscular disorders [4]. At the psychological level, it has been found that informal caregivers experience high levels of subjective burden, distress, and a wide variety of psychological disorders [5,13,21,24,25,26,27,28,29], as well as sleep problems [30,31,32]. In addition, as part of their role, caregivers also suffer other symptoms such as hostility, aggression, somatization, irritability, hallucinations, delusions, restlessness, and disinhibition [2,33,34,35]. Regarding social and financial troubles, informal caregivers usually expend the most of their time and economic resources in providing the patient with care; this leads to a reduction in leisure activities [5] and, probably, to social isolation. Accordingly, the quality of life of informal caregivers of patients with dementia is also seriously affected [26].

In the scientific literature, we have identified different kinds of interventions aimed to try to reduce the adverse impact of the caregiving-related duties on informal caregivers’ physical and psychological health as well as quality of life. While some of these interventions have been focused on improving the health-related quality of life of caregivers and care-recipients as well as on positively impacting the society, in general, and the care-recipient’s direct environment, in particular [36,37], a different series of interventions have been focused on reducing the experienced psychological distress and subjective burden in caregivers and care-recipients [11]. These interventions include support groups, respite care, training of person with dementia in developing caring tasks efficiently, and other multicomponent approaches [37,38,39,40]. In addition of these interventions, and according to the review by Ploeg et al., [9] there have also been a series of web-based interventions carried out, that have led to a reduction in a range of psychological symptoms in caregivers of patients with dementia [9]. According to some other literature reviews [11,41,42,43], some randomized controlled trials (RCT) have also been conducted for caregivers, even web-based interventions [9]. However, among these RCTs, relatively few RCTs were well-designed and, despite being clinically meaningful, they have only showed limited effects on various aspects of caregivers’ wellbeing, such as confidence, self-efficacy, depression or quality of life [44,45], or have reported inconsistent results for this population [46].

Previous research has also shown that exercise-based therapies are effective and feasible methods of improving health-related quality of life (physical and psychological) in patients [47] and caregivers [48,49,50,51,52,53]. These interventions have usually resulted in an increase of participants’ physical activity level [21,23,52]; have positive effects on their physical fitness [48]; and alleviate fatigue and subjective burden [43,50,52,54]. Other studies have found that exercise-based programs improve caregivers’ psychological health. The intervention programs of these studies included endurance exercise [51]; walking [54,55]; or yoga [56,57]. However, the results of these studies have to be considered cautiously, because only a few studies have designed the experiment on the basis of a RCT approach [23], and the available RCT-based studies used small sample sizes [58]. In addition to this limitation, the heterogeneity of the examined variables, such as duration of the intervention, physical exercise intensity, weekly dose of activity, as well as a broad range of used exercises, also make it difficult to generalize the results to a larger population [43,59,60]. 

On the other hand, a noteworthy fact is that informal caregivers usually have limited time to be engaged in preventive health-related activities, such as physical exercise [61]. Indeed, caregivers of relatives with dementia find it dificult to leave their home or demanding flexibility, due to their caregiving-related responsibilities. Moreover, the long distances to regular services of provision with the opportunity to do physical exercise or to conventional facilities, such as a gym, for example, when the caregiver lives in small and/or rural municipalities, do not help either caregivers get involved in physical exercises programs [62]. Thus, the home environment might be considered an advantageous context for the provision of an intervention that is based on physical exercise, since it means less time and even less financial resources for participants than the conventional community-based programs [23]. In addition, home-based interventions may also improve participants’ adherence to the intervention [63,64]. In this regard, previous research has shown that a home-based support program for non-professional caregiver generates more positive effects than its counterpart [65]. The main reasons are that the first kind of intervention offers to participants individual tailoring; they are less economically demanding and may be more easily accessed for many caregivers [9], especially for those who live in remote areas, such as rural regions [66].

To the best of our knowledge, there is a lack of studies assessing the cost-utility of exercise-based programs provide in home-based contexts for caregivers of relatives [67]. Moreover, there are no programs—within the public sanitary or welfare system—that provide informal caregivers with individual face-to-face programs at caregivers’ homes in Spain. In addition, there is a lack of RCTS-based interventions evaluating the cost-effectiveness of a home-based physical exercise program for informal caregivers. Therefore, RCTs of interventions that particularly target the needs of individual caregivers, and that evaluate the cost-effectiveness and impact of home-based physical exercise interventions on informal caregivers’ physical and psychological status—quality of life—are warranted [42]. Results of these interventions might contribute by being the basis for future health care policies that establish home-based health promotion services and facilitate financial support for these initiatives. In accordance with research gaps and expectations, the aim of the present study is to describe a protocol for the evaluation of the cost-effectiveness and the effects of an adapted home-based exercise program for female family caregivers on their health-related quality of life. Thus, several dimensions of health will be evaluated: health-related fitness variables (strength, endurance, flexibility, balance, and motor function); subjective burden and depression, psychological symptomatology and global health-related quality of life.

## 2. Material and Methods

### 2.1. Design

**Figure 1:**
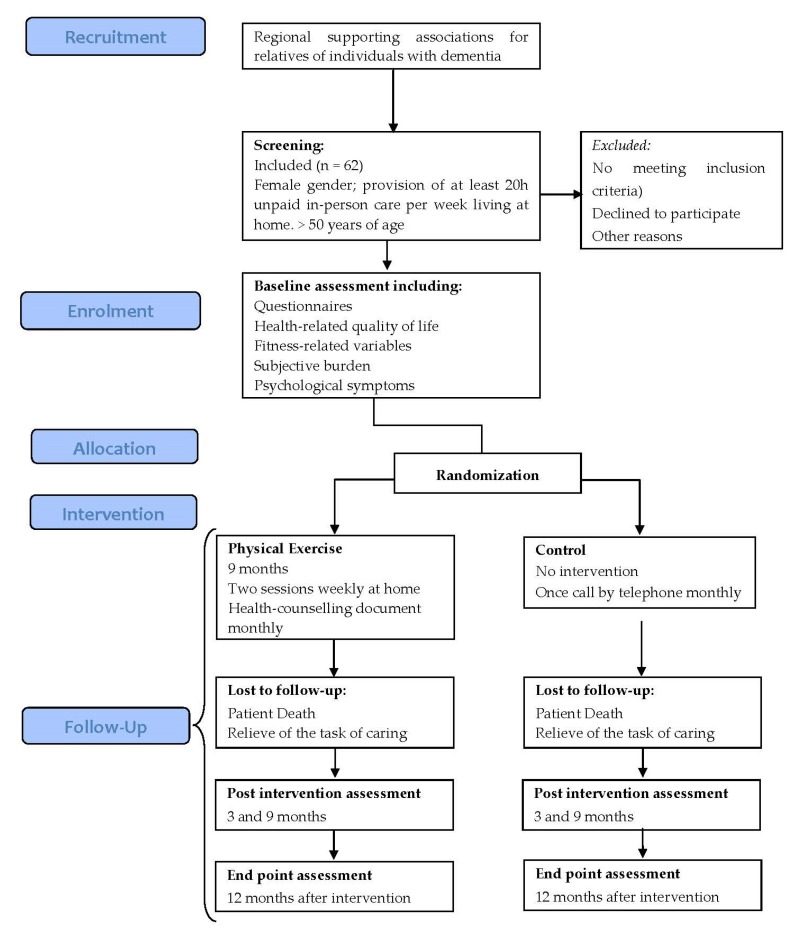
Design and participant flow chart.

A 9-month-long RCT will be performed to elucidate the cost-utility of a physical exercise program under the supervision of the main researcher of the current proposal, who is MSC in Health Economics and Pharma-economy. Participants will be randomly assigned to either the tailored home-based exercise group (intervention group) or the non-exercise group (control group). Assessment will be performed at baseline (pre-intervention assessment), 3 months, and 9 months after the intervention (post-intervention assessments) and 12 months after the intervention (post-intervention follow-up assessment); see Figure 1. The study is registered under clinical trial identifier ISCRCTN80414567, and will be conducted in accordance with the updates of the Declaration of Helsinki.

### 2.2. Participants

Participants will be recruited in cooperation with regional supporting associations for relatives of individuals with dementia. Potential participants will be identified and screened for eligibility in accordance with the study inclusion and exclusion criteria by trained members of the research team. The inclusion criteria will be: being a female caregiver of a relative with dementia; living at home with the patient with dementia; providing the relative with dementia with at least 20 hours of unpaid in-person care per week; being age 50 years or older; having no medical condition that could limit participation in a moderate-intensity exercise program; not having participated in any regular physical activity program, that is, having been engaged in less than two 20-minutes-long sessions of exercise per week during the 36 months prior to the intervention; not having changed medication or doses of medication for at least 3 months prior to study; and not having plans to move from the place of residence within the 12-month-long study. The exclusion criteria will be: having any external economic or personal support for caregiving of the patient with dementia. A written copy of the study protocol will be sent to all potential participants. In addition, at the first session of assessment, participants’ potential doubts will be solved by the member of research team. A signed informed consent will be requested to participate from all eligible participants prior to inclusion in the study.

#### 2.2.1. Sample Size Calculation

The required sample size will be estimated using the utility score of the Spanish version of the EQ-5D-3L questionnaire as primary outcome, and taken into account that this is an experimental study that will compare two groups with a significance level alpha = 0.05 and 80% of the statistical power that is required to detect a minimal clinically significant difference of 0.20 and SD = 0.12 [68].

On the other hand, we have also to consider the costs of the intervention added to the conventional care provided by the public health system, which will be 1680 € per caregiver. This amount will include the personal trainer’s salary, insurance, and 2 h/week travel-related costs.

A third aspect refers to the cost-efficiency ratio expressed as cost per quality-adjusted-life-years (QALYs). This will range between 10,000 and 50,000 € per QALYs [69], and we will use a conservative estimated sum = 30,000 € per QALYs. A cost-efficiency ratio = 0.21 corresponds to a 0.06 QALYs. In view of the above, a sample size of n = 50 caregivers distributed in two groups—control group and intervention group—is required to achieve a statistical power of 80% with a 95% of confidence interval, using an analysis of variance in parallel to parametric testing and anticipating a potential drop-out rate of 20%. In addition, in order to improve the confidence interval, a bootstrapping technique will also be used.

#### 2.2.2. Participants’ Randomization and Personal Data Management

Randomization will be carried out by an independent staff member, who will not be engaged in any other part of the assessment or intervention. For this purpose, a random computer algorithm will be used.

Regarding participants’ personal data, such as name, educational level, etc., the information of each participant will be individually encoded and saved in a locked file cabinet, kept in a separated room from the research laboratory, where the research team will work. Only some members of the research group will handle this information.

### 2.3. Intervention

The 9-month-long intervention, which will be an additional intervention to the conventional care provided by the public health system, as previously described, will include 2 home-based physical exercise 1-hour-long sessions per week for caregivers of relatives with dementia. These physical exercise sessions will be supervised by a physical trainer, who will have an academic degree in sport sciences, and who will be blind to the results of the assessments. Each session will be of home-based, individual and face-to-face nature. While the physical trainer and caregiver are performing the exercise session, the patient might stay at home, either sitting-down doing easy tasks (e.g., watching TV or playing with a pencil and paper-drawing or game task) or carrying out a craft that would be suitable for his/her level of deterioration.

The physical exercise intervention basically consists of aerobic exercises of moderate intensity, which means about 3 to 6 metabolic equivalents. In particular, each physical activity session will consist of: (1) 10-minutes-long warm-up activities that include slight movements of progressive intensity and easy walks and steps. (2) This will be followed by 10-minutes-long aerobic exercises at 60%–65% of maximal heart rate (HR_max_), which will be followed by 20-minute-long overall mobility and strength exercises using own body weight and other materials, which will be followed by a further 10-minute-long aerobic exercise at 60%–65% HR_max_, which will be finally followed by a 10-minute-long cool-down period that includes low-intensity exercises for participants to reach s state of calm.

In order to enhance the attractiveness and the positive effects of the exercises, supplementary materials, such as weights, dumbbells, and elastic bands will be used. During each physical activity session, participants’ heart rate will be monitored by means of a so-called individual running computer (Polar S625X, 1 Polar Electro Oy, Kempele, Finland).

During the 9-months-long intervention, and in order to reinforce other healthy habits in their daily lives apart from physical exercise, participants of the intervention group will be sent a health-counselling document once a month, in which the importance of the following issues will be addressed: adequate rest, safe positioning of the back during caring activities, and regular participation in daily social activities.

During the 9-months-long intervention period, caregivers of the control group will be instructed to continue their normal daily activities. They will also be instructed not to start any new physical exercise program to avoid influencing the study. However, the inclusion criteria of caregivers included no participation in any regular exercise program consisting in 20 minutes’ exercising in the previous 36 months. During the 9-months-long intervention period, participants of the control group will be contacted once a month by telephone in order to conduct an ad hoc standardized, non-exercise-related conversation with a member of the researcher team, in order to remind them of the conditions of the study.

### 2.4. Measures

Participants of both groups will be evaluated at home by trained members of the research team at baseline, 3 and 9 months after the intervention and at follow-up (12 months after the intervention). Each of the four evaluations will last 45 minutes. Prior to the baseline evaluation, and in order to apply a standardized evaluation protocol, the evaluators will be given a testing manual that has been ad hoc written by the research team. This manual contains the description and particular instructions for all assessment procedures. They will also participate in an assessment-training workshop that consists of 3 sessions, each 3–4 hours-long. This training workshop will be focused on improving evaluators’ skills to apply the protocol properly. The evaluators will be instructed to learn the particular procedures for implementing the questionnaires, and they will train their skills to perform the selected fitness tests and to proceed with the participants (e.g., how to prepare the session in a small room instead of a gym, avoiding interaction with the Alzheimer’s patient as previously described, or how to chat about other issues of society or needs of the caregiver to distract them from caring). This training will be supervised by the head of the research team.

All of the evaluation tools have been shown to have a moderate to high reliability and validity in samples of old people and caregivers.

#### 2.4.1. General Measures

Socio-demographic and other data from the caregivers will be compiled using a questionnaire that has been made ad hoc for this purpose. The compiled demographic data will include age, place of residence, marital status, number of co-residents, educational level, smoking and alcohol consumption habits, and level of physical activity. Other data will include the nature of the relationship with regard to the relative with dementia, as well as the number of years spent caring the relative with dementia.

Regarding the relatives with dementia, the number of years since diagnosis of dementia, as well as the functional status, will be evaluated by the trained member of the research team using the Barthel Index [70]. This 10-item measure assesses the care-recipient’s capacity to perform 10 basic activities of daily life, such as the ability to dress or bathe. Numerical scores are assigned according to whether the individual requires assistance to perform the task or whether he/she is able to do the activity independently. This will provide a quantitative estimation of the individual’s level of dependency, ranging from 0 to 100. Higher scores indicate a higher level of independence, and the opposite pattern is indicated by lower scores.

#### 2.4.2. Analysis of the Effects of the Intervention

As we have previously described, all participants will complete all evaluation procedures at baseline and at 3 and 9 months after the intervention and at follow-up (12 months after the intervention). During the 9-months-long intervention, data regarding participants’ use of public and private health services will be compiled. In particular, information will be compiled on hospital stays, medication and secondary and primary health-care appointments.

### 2.5. Data Statistical Analysis

To determine possible inter-group differences in baseline data, the mean values of all baseline variables will be analyzed using independent sample t-tests. Moreover, for continuous variables with a normal distribution, we will use the Kolmogorov–Smirnov test and the correction of Lilliefors. For categorical variables, in turn, we will use the Pearson’s chi-squared test. In addition, inter-group differences will be analyzed using the Mann–Whitney U-test for continuous variables and the χ^2^ test for categorical variables. An analysis of variance will be also performed to determine inter-group differences regarding potential changes in the measured variables over the 9-months-long intervention period. Possible confounding factors, such as age, subjective burden, caregiver-rated Barthel Index, and length of caregiving-history at baseline, will be treated as dependent covariates. To determine the magnitude of change, effect sizes will be calculated using the pre-post-control (PPC) design [71]. The use of repeated measurements in the PPC design allows data from each individual to be used as his or her own control data, which typically increases the power and precision of statistical tests [72]. To determine the variable that best describes and predicts changes in different parameters, an adjusted multiple regression analysis will be performed. For all tests, the significance level will be set at *p* < 0.05. All statistical calculations will be performed using SPSS 18.0 (SPSS Inc., Chicago, USA).

#### Cost Utility Analysis

This analysis will be performed in two steps. First, the incremental mean costs of the exercise-based program, and the mean increase in QALYs secondary to the program, will be estimated using a health-care perspective. Second, the incremental cost-effectiveness ratio for the intervention will be calculated by dividing the incremental costs by the incremental QALYs.

To report uncertainty due to sampling variation, the 95% confidence interval will be calculated using the non-parametric bootstrapping technique (1000 replicates re-sampled with replacement from treatment and control populations), and a cost-effectiveness acceptability curve will be plotted [73,74]. This curve shows the probability that the intervention is cost-effective compared with the alternative across the range of values that decision makers are willing to pay to achieve an additional QALY. The “investment ceiling” is the maximum possible level of spending, even under the assumption of unlimited funding availability. For the health-care system in Spain, the current year adjusted investment ceiling was set at a conservative €30,000/QALY (30000 to 50000 E/QALY is normally accepted).

Decision makers should compare this upper limit of acceptable payment with estimated incremental cost-effectiveness ratios, to determine whether a given treatment is cost-effective relative to the alternatives.

A range of sensitivity analyses will be performed to explore the robustness of the estimates and to determine how dependent the results will be on estimates of participants’ unit costs and efficacy. The first analysis will examine the influence of participation rate in the program, as this could influence productivity by affecting the number of participants per unit of time provided by the personal trainer. A second analysis will explore the effects of cost fluctuations that might be take place due to changes in the salary of the physical trainer, since this is a major source of variability in economic studies [75]. The time and cost of travel from the research center to the participant’s residence will also be calculated. Finally, the robustness of cost utility will be assessed by analyzing different possible scenarios, combining the influence of variations in staff salary, rate of participation, distance to the participant’s home, and effectiveness, ranging from the lowest to the highest limit of the 95% confidence interval.

## 3. Outcomes

### 3.1. Primary Outcomes

#### 3.1.1. Health-Related Quality of Life and Cost-Utility

The main purpose of the analyses is to assess the cost-effectiveness of the targeted intervention, which is an intervention added to the conventional care. Therefore, the primary outcome will be the Spanish time trade-off utility of EQ-5D-3L (EQ-5D-3L utility) in terms of QALYs. The EQ-5D-3L [76] will be used to assess the general health status and the five dimensions of health-related quality of life: (1) mobility, (2) self-care, (3) daily activities, (4) pain and discomfort, and (5) anxiety and/or depression of participants. Each dimension is rated using a 1 to 3 level scale, where 1 = no problem, 2 = some problems and 3 = extreme problems. Using a combination of these different dimensions, it is possible to identify a total of 243 possible health states. At the end of the screening, we will obtain a total score of utility that will range from 1 = fully functional quality of life to 0 = death [77]. Participants’ data regarding the QALYs compiled 9 months and 12 months after the intervention will be used to make comparisons between the intervention group and the control group. In this way, we will be able to find potential differential changes in the QALYs of the participants as a function of whether they were subjected to the intervention or not. For this purpose, participants’ health utility curves will be calculated and compared among participants of both sample groups. We include a 12-months-long follow-up period, following the recommendations of the National Health Service (NHS). To avoid bias, data will be adjusted for differences in baseline EQ-5D scores via regression analysis [78].

##### Cost-Utility: Cost Units

Since the study intervention is home-based, the intervention will have no direct impact on societal costs, such as travel expenses or the time spent by the caregiver at home, but the possible reduction of health visits to physician or nursery could vary. However, variation in travel costs (time and transport) of the initial visit to participants’ homes to explain the study and to evaluate the caregivers will be included in the sensitivity analysis. The economic analyses will be performed from a health service perspective, as recommended by the National Institute for Clinical Excellence (NICE) in the UK to inform decisions concerning health-care policy for an expensive condition. This may help to determine whether the addition of the program to the health system should be funded. The unit costs will be expressed in Euros (€) in the current year of intervention.

No adjustment will be required for changes in currency value over time, as the study will focus on effects observed over a period of ≤1 year. The costs of the program will be calculated on the basis of: university graduate salary levels; personnel costs for the running of the program; public-sector mileage rates; and the private external management costs of the program (insurance, basic portable sport materials, e.g., strings and step-equipment). Health-care prices (consultations, etc.) will be based on the official bulletin of the regional government of Extremadura. Drug prices will be obtained from the Spanish version of the Vademecum International [79].

#### 3.1.2. Subjective Burden

Participants will be also assessed using the Zarit Carer Burden Interview (ZBI) [80]. This 22-item self-report questionnaire examines the burden experienced by caregivers according to a rate scale which ranges from 0 to 88, where 0 indicates no burden and 88 indicates severe burden. The questionnaire addresses the functional, psychological, behavioral, and economic difficulties associated with the home care situation.

#### 3.1.3. Depression

Risk for depression will be assessed using the Short Form 15-item version of the Geriatric Depression Scale [81]. This 15-item self-report questionnaire is a reliable screening-tool for late-life major depression in the primary care setting. Scores range from 0 to 15. The presence of ≥5 symptoms indicates possible depression [82].

#### 3.1.4. Psychological Symptomatology

Psychological symptomatology will be measured using the Symptom Check List-90-R (SCL-90-R) [83]. This 90-item self-report questionnaire assesses the presence of psychological distress and a range of psychopathological symptoms. In particular, the SCL-90-R yields scores for nine primary symptom dimensions: somatization, obsessive-compulsive disorder, interpersonal sensitivity, depression, anxiety, hostility, phobic anxiety, paranoid ideation and psychoticism, as well as three indices of global distress. Each item is scored according to a 5-point Likert scale, which ranges from 0 = not at all to 4 = extremely. If the resulted global severity index is greater than or equal to a T score of 63th percentile or if the scores of two of those dimensions are greater than or equal to a T score of 63 or 75th percentile, this means that the respondent experiences severe symptomatology (positive case for caseness) [83].

#### 3.1.5. Fitness

- Weight, height, and the circumference of the waist and hip will be assessed according to the recommendations established by the European Council [84] for the calculation of body mass index (BMI) and waist / hip ratio (WHR).
- Handgrip strength will be assessed in both hands using a hand dynamometer (TKK; Tokyo, Japan). The mean value for the two hands will be used as the final outcome.
- Lumbar trunk muscle endurance will be assessed using two tests [85]. To evaluate flexor endurance, the subject will be asked to lie in a supine position and to raise the lower extremities with 90° flexion of the hip and knee joints. To evaluate extensor endurance, the subject will be asked to lie in a prone position while holding the sternum off the floor. During both procedures, the subject will be asked to maintain the original positions for as long as possible but without exceeding a 2-min time limit.
- Flexibility will be measured using the sit-and-reach test [86]. During this trunk flexion exercise, the distance between the tips of the fingers at the start and final positions will be recorded. The best result of three trials will be used as the outcome.
- Postural balance will be evaluated using the blind flamingo test [87,88,89]. Here, the barefoot subject stands on one leg with his/her eyes closed, while the other leg is flexed at the knee and held at the ankle by the hand of the same side. The chronometer is stopped whenever the subject does not comply with the protocol conditions. The number of trials required to remain static in this position for 30 s is measured. The outcome is expressed as the number of trials (=number of falls + 1).
- Lower extremity function will be assessed using the chair-stand test [90]. The subject sits in a standardized chair (0.43 m in height). The subject is asked to stand upright with his/her arms folded across the chest and then sit back down again. This is repeated 10 times, as quickly and as safely as possible. The best result of two trials (expressed in s) separated by 3 min is used as the outcome.

### 3.2. Secondary Outcomes

#### 3.2.1. Exercise Adherence

Each participant in the intervention group will be prescribed a total of 72 exercise sessions. Completion of the sessions across the 9-month-long intervention will be monitored by the supervising personal trainer. Exercise adherence rates during the intervention will be calculated as the percentage of the 72 prescribed exercise sessions.

#### 3.2.2. Potential Adverse Effects of the Intervention

Although it has been previously well documented that interventions that are based on physical exercises are beneficial for health, as this exercise program is provided at caregivers’ homes, the following potential adverse effects of the intervention have to be taken into account. First, physical harm or injuries. In this case, if the participant is severe injured, the personal trainer will recommend the caregiver to visit the doctor. If the caregiver is not able to continue the intervention because of the injury, he or she should withdraw the intervention. Second, caregivers may feel resentful while carrying out the physical exercises. Finally, the session of physical exercise might be interrupted because of caregivers’ behaviors.

## 4. Discussion

The provision of care to individuals with dementia is an unforeseen task that involves great efforts and represents a daily challenge for caregivers. This situation adversely impacts the physical, psychological, social, and financial well-being of caregivers [7,12,13,14,15]. In this sense, anxiety, depression, and subjective burden, and a multitude of other symptoms have been reported [2,5,13,24,28]. In addition, caregivers also show lower levels of fitness and higher rates of other physical disorders than non-caregivers [8,20,21,23]. As a consequence, this overburdened daily situation results in harmful effects on physical health and health-related quality of life [16,17,18,19,20] for caregivers. Hence, tailored interventions aiming to improve the health status of this population are warranted, as it is an important demand of society [36,37]. The health-related quality of life consists of several dimensions (e.g., mobility, self-care, doing daily activities, pain or discomfort and anxiety or depression) according to the EQ-5D-3L instrument described in methods. This health-related quality of life is worst in this population [20], especially in pain/discomfort, anxiety/depression and other psychological symptoms such as somatization, hostility or obsession-compulsion [91]. The intervention described in this article has been conceived to provide the targeted caregivers with a suitable support program aiming to improve the health-related quality of life, in response to the difficulties, such as lack of leisure-time or financial difficulties [58,92] that they usually have when it comes to participate in conventional exercise activity programs. Moreover, in order to increase the adherence to the intervention, it will be individually tailored, as previous research recommends [42]. In particular, the sessions will be provided at each caregiver’s home, and both the intensity and the types of the exercises will be adapted to each caregiver’s fitness level. Moreover, it is considered that the method of this study is robust and adequate to evaluate the effect of the intervention on family caregivers.

In the scientific literature, there is a wide range of supporting interventions for caregivers of relatives with dementia. According to this previous research, physical exercise interventions are considered as useful and feasible nonpharmacological strategies to improve the physical and psychological health of those caregivers [43,52,60]. However, there is a lack of tailored home-based physical exercise programs for such caregivers in Spain. Therefore, to the best of our knowledge, this is the first home-based physical exercise intervention that uses a controlled trial approach in this country for informal caregivers of relatives with dementia. 

On the other hand, several previous studies have suggested the implementation of home-based interventions, since these are considered as more effective in improving quality of life and reducing levels of distress [93]. The fact that an RCT approach will be used will enable us to provide scientific evidences on the efficacy and cost-effectiveness of a home-based physical exercise intervention to improve fitness and health-related quality of life for informal caregivers of patients with dementia. In addition, the outcomes of the present intervention could provide evidences for policymakers to strengthen home-based interventions health policies that might have implications to lessen the investments of financial resources in hospitalizations, health services and the intake of medication.

We are also interested in evaluating the cost-effectiveness of the physical exercise program on the whole health of caregivers. In this sense, the most widely used methodological approach is to measure the consequences in terms of quality-adjusted life years (QALYs) that will be gained by the caregivers after the intervention, because it can combine gains in quantity of life and gains in quality of life in a single metric [94]. For this reason, we have selected the EQ-5D-3L, a well-known tool for evaluating the cost utility of the targeted intervention. Additionally, as we have previously described, several standardized measures will be also used to assess fitness, health-related outcomes, and psychological symptoms among participants. These variables have been selected because, according to the available scientific literature, several previous research studies have frequently documented that informal caregivers usually report lower scores than other populations in fitness, health-related quality of life and psychological wellbeing [20,24,29,92]. This will generate feasible data concerning the effectiveness of the intervention for caregivers. In this regard, and according to previous research [58], it is expected that there will be an improvement on health-related quality of life, and an increase in fitness level, as well as a decrease of burden and psychological symptoms in the caregivers subjected to the present intervention. In fact, potential indirect positive effects could be reported for the care recipients [58], due to the improvements of their caregivers.

If the present study protocol contributes to confirming the effectiveness of the intervention for informal caregivers of relatives with dementia, this study protocol and the research study proposed here might be applied to other caregiver populations such as family caregivers of relatives with Parkinson’s Disease, stroke, cancer or even to caregivers who do not reside with the care-recipient, such as neighbors or friends.

Finally, we have to point out the main limitation of the present study. We are referring to the relatively small sample size, which may limit the generalization of the results to other contexts. However, the sample size is adequate for assessing the effectiveness of the targeted intervention, and future research efforts will have to determine the required adjustments for future studies, larger cohorts or other populations.

## 5. Conclusions

The present support intervention for caregivers of relatives with dementia may represent a tailored and feasible strategy to help those caregivers cope with the physical and psychological stress of acting as family caregivers of individuals with dementia. Moreover, obtaining confirmatory data may strengthen regional and national policies focused on improving home-based care services, and thus lessen patients’ demands of health-care resources. Finally, future research efforts could be developed by using other caregivers of patients with other pathologies, to benefit from the positive outcomes of this intervention.
